# Editorial: Diabetes and pregnancy: optimizing maternal and fetal outcomes through advanced technologies

**DOI:** 10.3389/fendo.2026.1872325

**Published:** 2026-05-18

**Authors:** Grenye O’Malley, Cassandra E. Henderson, Kristin Castorino

**Affiliations:** 1 Icahn School of Medicine at Mount Sinai, New York, NY, United States; 2 Rockwood Partners, LLC, New York, NY, United States; 3 Sansum Diabetes Research Institute, Santa Barbara, CA, United States

**Keywords:** algorithms, diabetes, gestational diabetes, pregnancy, technology

Diabetes in pregnancy is one of the most common and consequential complications of modern obstetric care. Normalizing glucose during pregnancy is not only paramount to reducing short term complications for mom and baby, but also to impact long-term metabolic health of the baby. The global prevalence of both type 2 diabetes mellitus (T2DM) in pregnancy and gestational diabetes mellitus (GDM) continues to rise, driven by increasing maternal age, higher rates of obesity, and declining metabolic health worldwide. As a result, hyperglycemia affects a rapidly increasing proportion of pregnancies. Leveraging diabetes technology for pregnancies complicated by diabetes, offers hope of more effective and individualized therapies to reduce risk, improve the health and offer novel strategies that could help combat key drivers of the global diabetes and obesity epidemic.

This expanding health crisis underscores a fundamental tension in healthcare: the need for accessible, scalable metrics that enable early and widespread detection, balanced against the need for recognition that pregnancy hyperglycemia is heterogeneous and demands precision and individualized approaches to management. Traditional screening paradigms, typically centered on mid-pregnancy oral glucose tolerance testing, may identify disease too late to fully mitigate adverse developmental exposures of pregnancy hyperglycemia. At the same time, advances in digital health, continuous glucose monitoring (CGM), and other wearable technologies now offer unprecedented detail of maternal physiology across the 24-hour day, presenting both opportunity and complexity.

This Research Topic addresses this paradigm. Together, they span the continuum from early prediction of GDM, to mechanistic insights into the intrauterine metabolic environment, to evaluation of advanced technologies and behavioral interventions aimed at improving maternal glycemia and perinatal outcomes. Collectively, they highlight how prediction, physiology, and digital health interventions can be integrated to improve outcomes in diabetes-affected pregnancies.

The first article explores cost-effective biomarkers to predict GDM in the first trimester. In a retrospective cohort of nulliparous women over 34 years old, Zhao et al. identified the readily identifiable biomarkers of as independent predictors of subsequent GDM when measured as early as 10–13 weeks’ gestation. Importantly, these markers are inexpensive, routinely collected, and globally accessible. The study proposes risk-stratified screening strategies for identification of hyperglycemia earlier in pregnancy in high-risk populations increasingly encountered in clinical practice.

Early detection of hyperglycemia is only meaningful if it improves outcomes. However, we are still trying to understand how maternal metabolic disturbances shape fetal development. Addressing this question, Villarroel et al. explored how cord blood provides a window into the intrauterine hormonal and metabolic milieu in pregnancies complicated by T2DM and GDM. By examining anti-Müllerian hormone (AMH), sex steroids, insulin, IGF-1, and adiponectin, the investigators demonstrate that maternal T2DM profoundly alters the fetal endocrine-metabolic environment. These findings reinforce the concept that diabetes in pregnancy is not merely a perinatal complication, but a determinant of offsprings’ future metabolic health.

Managing diabetes in pregnancy is difficult, and glucose monitoring guidelines are antiquated. Current pregnancy guidelines still require multiple fingerstick testing as gold standard for glucose monitoring, especially in T2DM and GDM. Further, best practice for managing diabetes in pregnancy emphasize frequent visits every 1–2 weeks, and many pregnant populations struggle with accessing care.

Two notable reviews evaluate the impact of CGM and mobile health interventions, highlighting the use of advanced technologies on maternal and fetal outcomes. Lai et al. analyzed 17 randomized controlled trials, demonstrating a significant reduction in neonatal intensive care unit admissions among pregnant women using CGM compared with standard self-monitoring of blood glucose. Trends toward reductions in large-for-gestational-age infants and neonatal hypoglycemia further support CGM’s clinical benefit, while the observed increase in small-for-gestational-age risk highlights a critical need for refinement of CGM-guided glycemic targets during pregnancy.

Complementing this work, Yang et al. analyzed mobile health interventions demonstrating that digital platforms integrating glucose monitoring, education, and communication can significantly reduce cesarean delivery, emergency cesarean delivery, and composite neonatal complications among women with GDM. Improvements in postprandial glucose levels reinforce the capacity of mobile health tools to support behavioral change and clinical management. Together, these reviews demonstrate that technology-enabled care models can meaningfully improve both maternal and neonatal outcomes.

The final two articles move beyond aggregate outcomes to explore emerging data on precision physiology and behavior using integrated wearable technologies, and perhaps the future of therapeutic interventions. Hallenbeck et al. linked CGM with wrist-worn activity monitors to examine the relationship between daytime physical activity and nocturnal glucose levels in individuals with pregnancy hyperglycemia. The findings of this feasibility study illustrate the complexity of behavioral–glycemic interactions and potential to pair continuous movement and glucose data to interrogate diurnal metabolic patterns. These insights set the stage for more granular, personalized interventions. The Time to Move randomized crossover trial protocol put forth by Ehrlich et al. formalizes an experimental approach to one such question: whether the timing of physical activity—morning versus evening—can be leveraged to optimize glucose control during pregnancy. By combining timestamped CGM data, objective physical activity monitoring, and detailed dietary assessments, this study exemplifies the next generation of pregnancy research that is data-dense, behaviorally informed, and precision-oriented.

Taken together, these studies illustrate a rapidly evolving field in which technology, physiology, and behavioral science converge to advance care for diabetes in pregnancy. There is a clear need for earlier and simpler detection of pregnancy-related dysglycemia, using cost-effective strategies that are scalable across diverse healthcare settings. Advancing care that drives improved outcomes requires a deeper understanding of the metabolic and hormonal environment of the fetus, particularly as maternal T2DM becomes increasingly prevalent. The integration of advanced technologies such as CGM, wearable devices, and mobile health platforms opens new avenues for precision and behavioral interventions.

These technological advances must be accompanied by explicit attention to health equity, ensuring that digital tools and advanced monitoring strategies are accessible to all populations, especially those at highest risk for diabetes in pregnancy, rather than exacerbating existing disparities.

This Research Topic points us toward a future in which pregnancy hyperglycemia is managed through earlier detection, continuous data streams, personalized interventions, and a more nuanced understanding of maternal–fetal metabolic interactions so that we can improve outcomes for mothers and the next generation.

